# The psychiatry of King Lear

**DOI:** 10.4103/0019-5545.31519

**Published:** 2007

**Authors:** Somasundaram Ottilingam

**Affiliations:** Former Superintendent (Retd.), Institute of Mental Health, Chennai, India

No other non-psychiatrist has been alluded to extensively and appreciatively quoted by psychiatrists as William Shakespeare: famous psychiatrists like Conolly, Maudsley, Bucknill and Crichton-Browne to name a few eminent 19^th^ century men. Shakespeare's characters like Othello and Ophelia have lent their names to the syndromes named after them. In the words of Bucknill:

‘Although for many years the dramas of Shakespeare have been familiar to the author, the extent and exactness of the psychological knowledge displayed in them, which a more diligent examination has made known, have surprised and astonished him. He can only account for it on one supposition, namely, that abnormal conditions of mind had attracted Shakespeare's diligent observation and had been his favourite study.’

In the tragedy of *King Lear* there are vivid descriptions of wandering, severely mentally ill persons in the personage of Tom O'Bedlam or “poor Tom” and disturbed raving madness in late life exemplified by Lear.

## Tom O'Bedlam

The Duke of Gloucester banishes his son and heir, Edgar, at the behest of Edmund, his scheming and covetous bastard son. Edgar now assumes the mantle of a wandering madman - Tom O'Bedlam - to hide his true self. In the various discourses by Edgar we get a clear description of the so-called Bedlamite beggars. They could be recognised from their odd mien, gestures and talk. Almost all the harmless psychotics were at large in the society, receiving alms and charity from the sympathetic community - which might be lacking at times, leading to starvation.

EDGAR  I heard myself proclaimed

And by the happy hollow of a tree

Escaped the hunt. No port is free, no place

That guard and most unusual vigilance

Does not attend my taking. Whiles I may scape

I will preserve myself; and am be thought

To take the basest and most poorest shape

That ever penury in contempt of man

Brought near to beast.

My face I'll grime with filth

Blanket my loins, elf all my hair in knots

And with presented nakedness outface

The winds and persecutions of the sky.

The country gives me proof and precedent

Of Bedlam beggars who, with roaring voices

Strike in their numbed and mortified bare arms

Pins, wooden pricks, nails, sprigs of rosemary;

And with this horrible object, from low farms

Poor pelting villages, sheep-cotes and mills

Sometimes with lunatic bans, sometime with

prayers

Enforce their charity. “Poor Turlygod, poor Tom!”

That's something yet! Edgar I nothing am.

Act 2, Scene 3, line 1.

Occasionally the menu of these pitiables could be:

EDGAR  Poor Tom, that eats the swimming frog, the toad

The tadpole, the wall-newt and the water

That in the fury of his heart

When the foul fiend rages

Eats cow-dung for sallets

Swallows the old rat and the ditch-dog

Drinks the green mantle of the standing pool

Who is whipped from tithing to tithing

And stock-punished and imprisoned

Who hath had three suits to his back, six shirts to his body

Horse to ride and weapon to wear

But mice and rats and such small deer

Have been Tom's food for seven long year.

Beware my follower. Peace, Smulkin; peace, thou

fiend!

Act 3, scene 4, line 123

Edgar bemoans his lot further:

EDGAR  Who gives anything to poor Tom?

Whom the foul fiend hath led through fire and through flame

Through ford and whirlpool, o'er bog and quagmire;

That hath laid knives under his pillow and halters in his pew;

Set ratsbane by his porridge; made him proud of heart

To ride on a bay trotting horse over four-inched bridges

To course his own shadow for a traitor. Bless thy five wits!

Tom's a-cold. O, do de, do de, do de.

Bless thee from whirlwinds, star-blasting and taking!

Do poor Tom some charity, whom the froul fiend vexes.

Act 3, scene 4, line 50

In spite of his poor mental health King Lear has words of sympathy for the poor Toms:

LEAR  Poor naked wretches, wheresoe'er you are

That bide the pelting of this pitiless storm

How shall your houseless heads and unfed sides,

Your looped and windowed raggedness, defend you

From seasons such as these? O, I have ta'en

Too little care of this!

Act 3, scene 4, line 28

Hearing imaginary voices is another hallmark of serious mental illness, well-known to the laity:

EDGAR  The foul fiend haunts poor Tom in the voice of a nightingale.

Hoppedance cries in Tom's belly for two white herring

Croak not, black angel; I have no food for thee.

Act 3, scene 6, line 30

The causation of mental illness has remained an enigma from Antiquity to the current day. Possession by spirits, either unholy or holy, is the favoured one of many attributed causes as the source of mental illness; poor Tom is no exception:

EDGAR  Five fiends have been in poor Tom at once:

As Obidicut, of lust; hobbidedence, prince of darkness;

Mahu, of stealing; Modo, of murder;

Flibbertigibbet, of mocking and mowing,

Who since possesses chambermaids and waiting-women.

Act 4, scene 1, line 56

EDGAR  Frateretto calls me and Nero is an angler in the lake of darkness. Pray, innocent, and beware the foul fiend.

Act 3, scene 6, line 8

EDGAR  This is the foul Flibbertigibbet.

He begins at curfew and walks till first cock.

He gives the web and the pin

Squinies the eye and makes the harelip;

Mildews the white wheat and hurts the poor creature of earth.

S'Withold footed thrice the ‘old:

He met the nightmare and her ninefold;

Bid her alight

And her troth plight -

And aroint thee, witch, aroint thee!

Act 3, scene 4, line 110

Edgar, malingering the role of seriously mentally deranged schizophrenic at large, overdoes his part to impress the onlookers, a common stratagem put on by malingerers. It appears as if Shakespeare has foreseen the poor Toms walking the streets of London and New York after de-institutionalisation has come into vogue in present times.

## KING LEAR'S MADNESSTH

GLOUCESTER  O ruined piece of nature!

Act 4, scene 6, line 135

EDGAR  O thou side-piercing sight!

Act 4, scene 6, line 85

It is extremely difficult to ascertain where in the play *King Lear* the hero becomes mad. The abnormal behaviour, the extreme irritability, the exhibition of disinhibited thoughts may be the harbinger of psychosis or his premorbid traits. When the Duke of Kent pleads lenience in Lear for Lear's youngest daughter Cordelia, he banishes them from his realm and explodes:

Peace, Kent!

Come not between the dragon and his wrath.

I loved her most and thought to set my rest

On her kind nursery.

Hence and avoid my sight!  [*To Cordelia*]

Act 1, scene 1, line 120

When Goneril, his eldest daughter, urges him to reduce his retinue of knights and attendants, Lear raves against her:

Degenerate bastard, I'll not trouble thee;

Act 1, scene 4, line 242

Detested kite, thou liest!

Act 1, scene 4, line 251

Hear nature; hear, dear goddess; hear!

Suspend thy purpose, if thou didst intend

To make this creature fruitful.

Into her womb convey sterility;

Dry up in her the organs of increase;

And from her derogate body never spring

A babe to honour her! If she must teem,

Create her child of spleen, that it may live

And be a thwart disnatured torment to her.

Let it stamp wrinkles in her brow of youth,

With cadent tears fret channels in her cheeks,

Turn all her mother's pains and benefits

To laughter and contempt, that she may feel

How sharper than a serpent's tooth it is

To have a thankless child! Away, away!

Act 1, scene 4, line 264

Extreme hatred exhibited by the father exemplified in the vituperative curses in the presence of the son-in-law seems to be very inappropriate behaviour to put it mildly.

Lear expresses the foreshadowing of his total disintegration in many statements:

Does any here know me? This is not Lear:

Does Lear walk thus, speak thus? Where are his eyes?

Either his notion weakens, his discernings

Are lethargied - Ha! waking? ‘tis not so.

Who is it that can tell me who I am?

Act 1, scene 4, line 214

O let me not be mad, not mad, sweet heaven!

Keep me in temper; I would not be mad!

Act 1, scene 5, line 40

O Lear, Lear, Lear!

Beat at this gate that let thy folly in

[*striking his head*]

And thy dear judgement out!

Act 1, scene 4, line 260

Perplexity and fear of approaching insanity are not uncommon in persons ending up with serious mental disorder like dementia.

Lear's frank madness is obvious to others and to himself. When Cordelia hears of his wanderings on the heath during the severe storm she exclaims:

Alack, ‘tis he! Why, he was met even now

As mad as the vexed sea, singing aloud,

Crowned with rank fumiter and furrow-weeds,

With hardocks, hemlock, nettles, cuckoo-flowers,

Darnel and all the idle weeds that grow

In our sustaining corn. A century send forth;

Search every acre in the high-grown field,

And bring him to our eye.

Act 1, scene 4, line 1

On seeing Lear's festooned appearance Edgar laments:

O thou side-piercing sight!

Act 4, scene 6, line 85

It is interesting to note that the flowers decorating King Lear have a particular medicinal value in treating insanity. On hearing Lear's ramblings Gloucester cries:

O ruined piece of Nature!

Act 4, scene 6, line 135

In the rambling speeches of King Lear we could perceive his vivid visual hallucinations, punning and rhyming and the delusions of grandeur:

Contending with the fretful elements,

Bids the wind blow the earth into the sea,

Or swell the curled waters ‘bove the main,

That things might change or cease; tears his white hair,

Which the impetuous blasts with eyeless rage

Catch in their fury and make nothing of;

Strives in his little world of man to out-storm

The to-and-fro-conflicting wind and rain.

This night, wherein the cub-drawn bear would couch,

The lion and belly-pinched wolf

Keep their fur dry, unbonneted he runs,

And bids what will take all.

Act 3, scene 1, line 4

Nature's above art in that respect. There's your press-money.

That fellow handles his bow like a crow-keeper:

Draw me a clothier's yard. Look, look, a mouse!

Peace, peace; this piece of toasted cheese will do't. There' my gauntlet;

I'll prove it on a giant. Bring up the brown bills.

O, well flown, bird; i'th' clout, i'th' clout: hewgh!

Give the word.

Act 4, scene 6, line 85

He conducts a mock trial for adulterers:

Ay, every inch a king!

When I do stare, see how the subject quakes.

I pardon that man's life. What was thy cause?

Adultery?

Thou shalt not die. Die for adultery? No!

The wren goes to 't and the small gilded fly

Does lecher in my sight.

Let copulation thrive: for Gloucester's bastard son

Was kinder to his father than my daughters

Got ‘tween the lawful sheets.

To ‘t, luxury, pell-mell! For I lack soldiers.

Act 4, scene 6, line 108

Lear misidentifies the Duke of Gloucester as his daughter Goneril with a white beard.

When Cordelia meets Lear after his rescue from the heath he fails to recognise her:

CORD Sir, do you know me?

LEAR  You are a spirit, I know; when did you die?

Act 4, scene 7, line 48

Lear expresses his perplexity and confusion in his own words; his cognitive difficulties are easily discernible to him:

Pray do not mock me;

I am a very foolish old man,

Fourscore and upward, not an hour more nor less;

And, to deal plainly,

I fear I am not in my perfect mind.

Me thinks I should know you and know this man,

Yet I am doubtful: for I am mainly ignorant

What place this is; and all the skill I have

Remembers not these garments, nor I know not

Where I did lodge last night.

Act 4, scene 7, line 60

LEAR  Am I in France?

KENT  In your own kingdom, sir.

Act 4, scene 7, line 75

Another instance of the psychotic behaviour of King Lear is seen in the mock trial of his daughters *in absentia*:

LEAR  I'll see their trial first. Bring in their evidence.

[*to Edgar*] Thou robed man of justice, take thy place;

[*to the Fool*] And thou, his yokefellow of equity,

Bench by his side.

[*to Kent*] You are o'th' commission;

Sit you too.

Act 3, scene 6, line 35

LEAR  Arraign her first; ‘tis Goneril. I here take my oath

Before this honourable assembly,

She kicked the poor king, her father.

Act 3, scene 6, line 45

LEAR  And here's another, whose warped looks proclaim

What stone her heart is made on. Stop her there!

Arms, arms, sword, fire! Corruption in the place!

False justicer, why hast thou let her scape?

Act 3, scene 6, line 52

## CONCLUSION

Madness in *King Lear* continues it's spell over the psychiatrists of the 20^th^ and 21^st^ centuries. Andreasen[1] makes a diagnosis that “Lear's madness can be explained in part as the development of a psychotic disorganization precipitated by severe stress in an elderly man already showing some signs of senile organic brain disease.” Therefore, in her interpretation, “Lear has a mild organic brain syndrome that develops under stress into a reactive psychosis.”

In his 1983 article, Kail[2] takes an interesting excursion into the history of psychiatry, as it relates to Shakespeare and also diagnoses in Lear “a case of progressive senile dementia” that is “accompanied by attacks of what could be described today as acute mania, as demonstrated by his faulty judgment, disorientation and irrational behavior.”

Colman[3] established for Lear a diagnosis of brief reactive psychosis with a background of organic mental disorder, perhaps of a vascular origin, exemplified by the King's visual hallucinations and an intimation of a stroke just before Lear's death.

Trethowan thinks that Lear was actually depressed, a victim of “involutional melancholia”.

According to Truskinovsky[4] the case of Lear warrants the diagnosis of bipolar I disorder, most recent episode manic, severe with psychotic features. The manic episode was primary and the psychosis developed on its background, provoked by the increasing agitation and physical exertion.

It's a wonder how Shakespeare's characters fit so well into the categories of DSM IV and ICD 10. We can only join Ben Jonson when he says of Shakespeare “He was not of an age, but for all time.”

## RECOMMENDED READING

1. Bucknill JC. The psychology of Shakespeare. Three Hundred Years of Psychiatry. Hunter R, Macalpine I, editors. Oxford Press: London; 1964. p. 1064-7.
2. Donnelly J. Incest, ingratitude and insanity: Aspects of the psychopathology of King Lear. Psychoanalytic Rev 1953;40:149-55
3. Feder L. Madness in Literature. Princeton University Press: 1980.
4. Ferriar J. Medical histories and reflections. Three Hundred Years of Psychiatry. Hunter R, Macalpine I, editors. Oxford Press: London; 1964. p. 543
5. Hunter R, Macalpine I, editors. Three Hundred Years of Psychiatry. Oxford Press: London; 1964. p. 5.
6. Kraepelin E. One hundred years of psychiatry. Philosophical Library: New York; 1964. p. 20.
7. Porter R, editor. The faber book of madness. Faber and Faber: London; 1991.
8. Somerville H. Madness in shakespearean tragedy Richards Press: London; 1929.
9. Trethowan WH. Psychiatry and the seven ages of man. J R Soc Med 1988;81:189-93
